# Interventions for Indigenous Peoples making health decisions: a systematic review

**DOI:** 10.1186/s13690-023-01177-1

**Published:** 2023-09-27

**Authors:** Janet Jull, Kimberly Fairman, Sandy Oliver, Brittany Hesmer, Abdul Kareem Pullattayil, Not Deciding Alone Team

**Affiliations:** 1 School of Rehabilitation Therapy, Queen’s University, Kingston, ON Canada; 2 Ottawa Hospital Research Institute, University of Ottawa, Ottawa, ON Canada; 3 Institute for Circumpolar Health Research, Northwest Territories, Yellowknife, Canada; 4 University College London, London, UK; 5 Queen’s University, Kingston, ON Canada

**Keywords:** Indigenous, First Nations, Inuit, Métis, Urban Indigenous, Shared decision making, Health, Equity, Systematic review, Framework synthesis, Integrated knowledge translation, Coproduction

## Abstract

**Background:**

Shared decision-making facilitates collaboration between patients and health care providers for informed health decisions. Our review identified interventions to support Indigenous Peoples making health decisions. The objectives were to synthesize evidence and identify factors that impact the use of shared decision making interventions.

**Methods:**

An Inuit and non-Inuit team of service providers and academic researchers used an integrated knowledge translation approach with framework synthesis to coproduce a systematic review. We developed a conceptual framework to organize and describe the shared decision making processes and guide identification of studies that describe interventions to support Indigenous Peoples making health decisions. We conducted a comprehensive search of electronic databases from September 2012 to March 2022, with a grey literature search. Two independent team members screened and quality appraised included studies for strengths and relevance of studies’ contributions to shared decision making and Indigenous self-determination. Findings were analyzed descriptively in relation to the conceptual framework and reported using guidelines to ensure transparency and completeness in reporting and for equity-oriented systematic reviews.

**Results:**

Of 5068 citations screened, nine studies reported in ten publications were eligible for inclusion. We categorized the studies into clusters identified as: those inclusive of Indigenous knowledges and governance (“Indigenous-oriented”)(*n* = 6); and those based on Western academic knowledge and governance (“Western-oriented”)(*n* = 3). The studies were found to be of variable quality for contributions to shared decision making and self-determination, with Indigenous-oriented studies of higher quality overall than Western-oriented studies. Four themes are reflected in an updated conceptual framework: 1) where shared decision making takes place impacts decision making opportunities, 2) little is known about the characteristics of health care providers who engage in shared decision making processes, 3) community is a partner in shared decision making, 4) the shared decision making process involves trust-building.

**Conclusions:**

There are few studies that report on and evaluate shared decision making interventions with Indigenous Peoples. Overall, Indigenous-oriented studies sought to make health care systems more amenable to shared decision making for Indigenous Peoples, while Western-oriented studies distanced shared decision making from the health care settings. Further studies that are solutions-focused and support Indigenous self-determination are needed.

**Supplementary Information:**

The online version contains supplementary material available at 10.1186/s13690-023-01177-1.

## Background

Systemic racism continues to challenge the resilience and undermine the self-determination of Indigenous^1^ Peoples [1–3] by severely restricting their capacity to participate and innovate for better health outcomes. Indigenous Peoples demonstrate tremendous resilience, self-determination, and capacity to innovate [4–6]. Despite these strengths, there are global disparities in health between Indigenous and non-Indigenous Peoples [7–11]. For example, the health of urban Indigenous, Inuit, First Nations, and Métis populations in Canada have been described as poor, with shorter life expectancies, and higher rates of illness, injury, and mental health issues when compared to general populations [10, 12, 13], a pattern that is evident in other colonial societies such as the United States, Australia and New Zealand [14]. Additionally, Indigenous Peoples consistently report poor experiences in health and social systems, that undermine access and uptake of services [15–17]. These disparities are the result of socially produced structures that are perpetuated by and directly related to colonialism [8]. The result is “health inequities”, defined as preventable, systematic, and socially produced differences in health between and within populations [7]. Indigenous Peoples must have opportunities to be central to and participate in health care that meets the needs they identify as important, that is, health care that is person-centred [18].

Shared decision making is a central feature of person-centred care [19, 20]. It is a process that engages people who are personally experiencing a health issue (“patients”), and their families, with health care providers to make decisions about screening, treatments, or management of chronic conditions [21]. We use the term ‘health care provider’ to refer to all people who are engaged in actions whose primary intent is to improve health, including both health care professionals (e.g. audiology, speech language pathology, dentistry, medicine, nursing, midwifery, occupational, physiotherapy) and health support workers (e.g. peer support worker, lay health worker) [22]. Standard care provides patients with evidence-based information about their health choices. With shared decision making, the patients' informed preferences, values and beliefs, along with clinical evidence, are also considered in making health decisions [23, 24] There are approaches and tools such as decision coaching and patient decision aids, that can support people to work with their health care providers and participate in their health decisions [25, 26]. Studies have shown that shared decision making improves patient outcomes and experiences [27], improves the experiences and effectiveness of health professionals in their communication with patients [28], and may optimise costs in health care [29]. Shared decision making has also been found to benefit those who are more likely to experience disadvantage in health systems [30].

There is a growing body of work about how shared decision making may enhance opportunities for Indigenous Peoples to participate in their health decision making; however, little is known about the scope of the literature regarding approaches and tools as strategies to support shared decision making with Indigenous Peoples. A systematic review, published in 2013, focused on interventions for Indigenous Peoples making health decisions and identified one study that reported a shared decision making strategy [31]. The literature search for the systematic review was conducted with no start date limitation, i.e., from the earliest data sources on each database, e.g., 1947 or earlier, and up to 16 September 2012. That review found many studies that were aimed at educating Indigenous participants to comply with particular health behaviours, rather than engaging them in a process of making shared decisions with health care providers [31].

Since the 2013 review, there have been important guiding documents that assert the right to self-determination of Indigenous Peoples, and promote actions to not perpetuate or reproduce existing colonial structures in society [32–34]. As well, these documents explain why it is important to conduct research to advance self-determination, in ways that Indigenous Peoples themselves identify as strengths-based, respectful and inclusive. In addition, there are a growing number of research guidelines for the conduct of research done with Indigenous Peoples [35–38]. The results of these research guidelines are contributions to research outcomes in the areas of balancing individual and collective rights, upholding ethical principles and community-driven research that upholds self-determination [39]. Shared decision making may be one way to address the health inequities experienced by Indigenous Peoples. The research to develop and evaluate shared decision making strategies must be done in ways that advance self-determination and are identified as respectful and inclusive.

We are a team of Inuit and non-Inuit members of service providers and academic health care researchers who are active in health care systems that provide services to communities in the Qikiqtani (Baffin) region of Nunavut and in Ottawa, Ontario. In a research project called “Not Deciding Alone”, we have been working to enhance opportunities for Inuit to participate in decisions about their health care. The purpose of Not Deciding Alone is to promote Inuit self-determination in research processes [38] in the development of interventions to support shared decision making in health systems. Our work is conducted with Inuit service organizations and community member partners and described in detail elsewhere [17].

Our team decided that it is important to determine the state of the research evidence and learn from interventions that support shared decision making with Indigenous Peoples. We decided to conduct a systematic review of the international literature (“the review”).

The purpose of the review is to identify interventions to support Indigenous Peoples making health decisions. The objectives are to a) synthesize evidence from studies focused on the development and/or testing of interventions to support Indigenous Peoples making health decisions, and; b) identify factors, such as barriers and facilitators, that impact the use of interventions to support Indigenous Peoples making health decisions.

## Methods

The Not Deciding Alone team consists of an Inuit and non-Inuit Steering Committee, who guide the work and includes an Elder, and academic research members who operationalize research tasks (JJ, KF, BH) (www.notdecidingalone.com) To complement the skills of the Not Deciding Alone team in the conduct of our review, we engaged two additional non-Indigenous research team members with expertise in library science (AD) and framework synthesis methods (SO). The review team (“the team”), all authors on this paper, between them hold knowledge and/or experience in the areas of Inuit societal values, Inuit and Indigenous health issues, shared decision making tools and approaches, knowledge translation, systematic review methods, framework synthesis, library sciences, collaborative research approaches with Inuit and Indigenous Peoples, qualitative and quantitative research methods. The first author (JJ) identifies as a non-Indigenous settler scholar of Euro-Canadian descent, and the second author (KF) identifies as Nunavummiut. The team shares concerns about Inuit access and uptake of health services, and experiences of Inuit in health systems. Our aim is to conduct research from within equitable partnerships that prioritize Inuit knowledge and experience, to ensure that Inuit communities are the primary benefactors of our work.

**Fig. 1:**
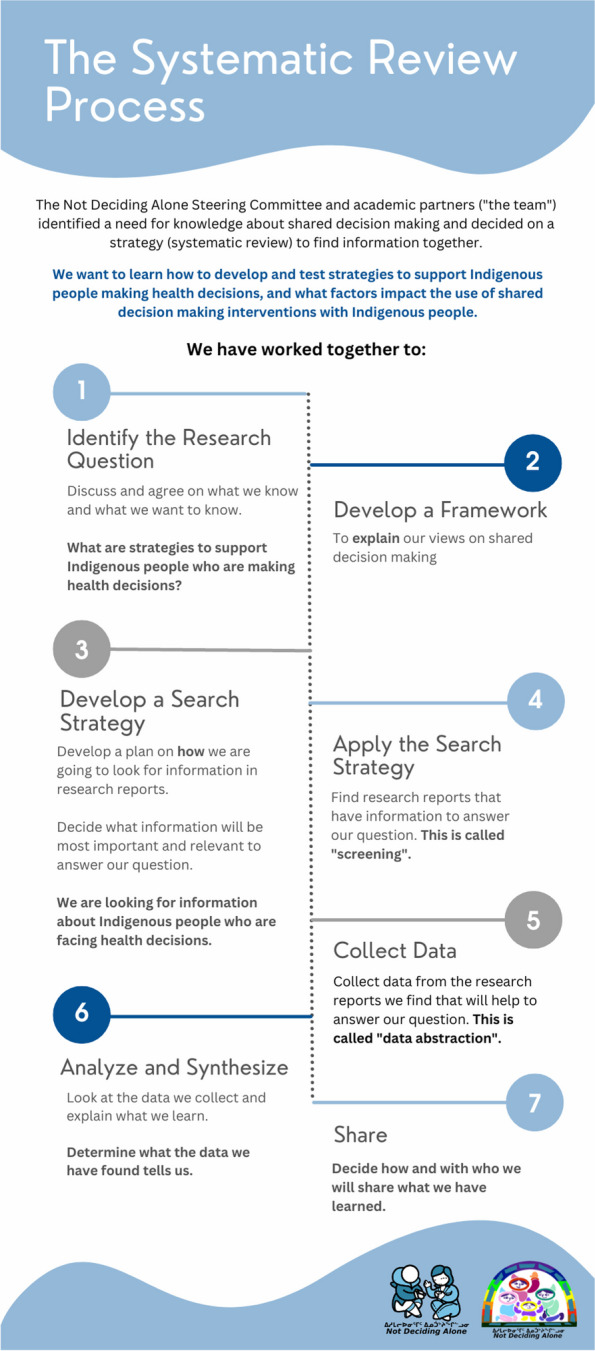
The systematic review process

To ensure transparency and completeness in our work, we used the Enhancing Transparency in Reporting the Synthesis in Qualitative Research (ENTREQ) [40] (Supplementary file 1) and the Preferred Reporting Items for Systematic Reviews and Meta-Analysis statement extended for equity (PRISMA-Equity) [41] (Supplementary file 2). We organized our study with the National Inuit Strategy on Research (NISR) [38]. We conducted our research to align with the views expressed by Inuit in community consultations, and with a focus on a principled approach to research [35, 42]. The principles of Inuit Qaujimajatuqangit guide a strengths-based approach to research and promotes Inuit self-determination and self-reliance. Inuit Qaujimajatuqanigit centre on collaborative decision making and working together for the common good [43, 44]. Previous work has shown the relevance and importance of centring on and promoting Inuit worldviews in both the research processes [45] and in the development and use of shared decision making research products [46]. Our study uses an integrated knowledge translation (KT) approach [47, 48] and is structured to support coproduction with study governance and collaborative conduct by those who will use or be impacted by the research [49]. The Steering committee members of the team were engaged with research team members throughout the entire review process to conceptualize, guide, reflect on, and amend or approve each step (Fig. 1).

### Theoretical framework

There are systematic reviews that demonstrate the benefits of frameworks to organize thinking about relationships between concepts [50]. We used a framework to organize concepts important to shared decision making in Indigenous contexts. To understand interventions for Indigenous Peoples making health decisions requires attention to knowledges that are not yet evident within Western-oriented health and social care frameworks [16]. We consider people and communities to be nested within broader structural and contextual contexts [8, 51, 52], and conceptualize shared decision making as a highly relational process.

Here, we explain our perspective on shared decision making, in relation to others’ work. Interventions to support shared decision making processes are described in frameworks and models that reflect shared decision making concepts, support a relational approach to shared decision making, and are based on research. The Ottawa Decision Support Framework (ODSF) describes decision support interventions, such as decision coaching and patient decision aids, as useful to address patients’ decisional needs [53, 54]. The Interprofessional Shared Decision Making (IP-SDM) model broadens the perspective of shared decision making beyond the patient-practitioner dyad, and positions shared decision making to be operating at the levels of patients, health care provider teams, and the health care system [55, 56]. Likewise, the Making Informed Decisions Individually and Together (MIND-IT) model describes the multiple-roles in shared decision making in health care systems and identifies factors and roles of patient and health care providers, and other stakeholders, that impact patient and clinician reasoning about health decisions [57, 58]. Another framework called “Aspects of patient involvement”, relates the complexity of involvement in decision making and patient relationships with health care providers [59]. Finally, the Medicine Wheel Framework was developed with an urban Indigenous community and describes their perspectives on shared decision making, with their roles and perceptions of health care providers’ roles within the shared decision making process [60]. The Medicine Wheel Framework is used to explain the relational nature of shared decision making, and the role of shared decision making in culturally safe care [60].

**Fig. 2:**
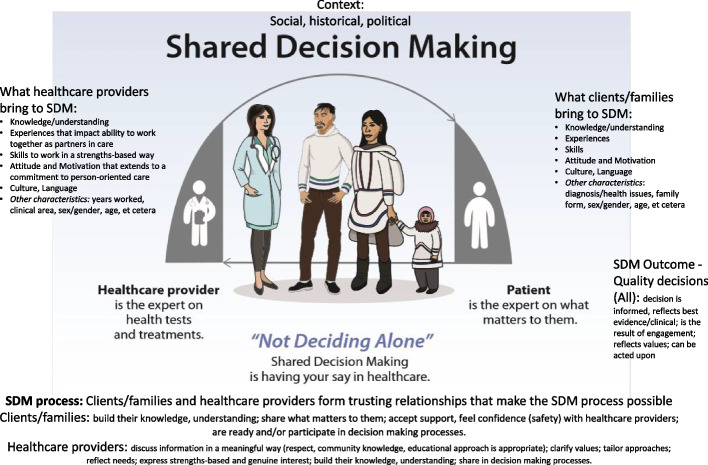
The shared decision making process framework

The pre-existing frameworks and models focus on interactions between patients, health care providers and health systems. Our team developed the Shared Decision Making Process Framework, to organize concepts important to Indigenous Peoples and shared decision making (“conceptual framework”). The conceptual framework is based on understandings of shared decision making by our team [25], our learning in collaboration with Inuit community partners, and incorporates concepts from the Indigenous and non-Indigenous shared decision making frameworks and models discussed here. The purpose of the conceptual framework is to provide a foundation for reflection on concepts important to shared decision making processes. The conceptual framework describes the context and people involved, and their influence on shared decision making processes and practice. (Fig. 2; see also Supplementary file 3).

Our team's perspective on shared decision making reflects processes that uphold relationality, mutual learning, and co-production of knowledge. Scholars have identified the importance of resolving oppression of Indigenous Peoples from within partnerships [61]. Our intent is to identify factors that impact the use of shared decision making as a strategy to correct imbalances of power within health systems. Our perspective centres on the need to align Indigenous Peoples as equal partners within health systems, and to support opportunities for participation in health decisions.

### Study inclusion and exclusion criteria

We framed the study inclusion and exclusion criteria in a systematic manner, using the elements of a clinical question and include population, intervention, comparator, and outcome and study description (PICOS) [62]. We included primary studies published in peer-reviewed journals that met our inclusion criteria, with no restrictions on the types of studies to be included: 1) Populations identified as Indigenous and making a health decision for themselves and/or a family member, 2) Interventions to influence making a health decision, 3) Any comparators for intervention studies examining effectiveness and not applicable for qualitative studies, 4) Outcomes that report on factors that impact the attributes of the decision or decision process, 5) Any study design. We excluded any papers for which full text was not available, and non-peer reviewed studies (editorials, commentaries, letters, dissertations).

### Data searches

We developed the search strategy protocol with the academic librarian team member (AP), after consultation with subject experts on health, decision-making and Indigenous and health research, and based on an earlier study [31]. The search strategy included subject headings and keywords for: the concepts of Indigenous identity combined with the concepts of informed or shared decision making, consumer health or health literacy. The librarian conducted searches for our team in major databases that cover the subject matter and without language restriction. The database selection includes: OVID MEDLINE, Global Health, CINAHL, Scopus, Native Health Database, Arctic Science and Technology Information System (ASTIS) database, Arctic Health, Circumpolar Health bibliographic database, and WHO’s Global Index Medicus. Due to the limited data available prior to 2012, we have focused our search to the previous 10 years September 17, 2012 to March 17, 2022. We did not limit by language. To supplement the database searches, we chose to conduct a Google Scholar search to cover grey literature as well as to identify relevant studies that have cited each of the included studies (forwards searching) and reference lists of included studies (backwards searching). We contacted experts on shared decision making and authors of included studies to identify other relevant studies (Supplementary file 4).

All screening was conducted by two authors (JJ, BH) using Covidence [63], with the oversight of a third author (KF) and discussion among all three authors about studies for which there was uncertainty about inclusion or exclusion. First, a title and abstract screen was conducted by two authors independently to determine study relevance to the overall objective of the systematic review and following the eligibility criteria. All studies identified as “included” and “unsure” were retained for full text screening; only studies excluded by both authors were excluded. The full text screening was based on the inclusion/exclusion criteria. During full text screening, the rational for exclusion was documented and final decisions confirmed with the team.

### Study quality: reflections on strengths and relevance

Following team discussions, we chose to focus on “quality” as a reflection on the strengths and relevance of included studies’ contributions to shared decision making and Indigenous self-determination. Our team wanted to: prompt reflection on study practices to develop and/or test interventions; identify factors that impact the use of interventions to support health decision making; and, include the knowledge of those who use or are the focus of the shared decision making intervention. We used tools intended for use with heterogeneous Indigenous studies, and studies conducted with Aboriginal and Torres Strait Islanders [64, 65]. Using the Well Living House quality appraisal tool [64], each document was scored in three domains: 1) community relevance, 2) rigour of study methodology, and 3) strength of evidence. Each included study was rated out of a total possible score of 12, within a range: low = 6–7, med = 8–9, high = 10–12. For Indigenous self-determination we used a reporting guideline developed to improve the quality of published health research and health outcomes, called the Consolidated criteria for strengthening reporting of health research for Indigenous Peoples (CONSIDER statement). We used the CONSIDER statement to guide reflection on whether the criteria of research priorities, governance, relationships, methodologies, capacity, and dissemination are reported in the studies [65]. Two authors (JJ, KF) independently assessed the included studies, then the research team (JJ, KF, SO, BH) engaged in discussion and reflection that extended to the whole team (JJ, KF, SO, BH, with the guidance and approval of the Not Deciding Alone Team). The reflections on strengths and relevance of each study and any discrepancies in assessment between the team members was discussed and final decisions agreed upon and reported.

### Data abstraction

We used our conceptual framework to reflect concepts related to shared decision making and to guide data abstraction and analysis of included studies. We included criteria for general study information from reporting guidelines to improve completeness in reporting of interventions [66], and research conduct [65].

Two authors (JJ, BH) conducted a pilot test of the data collection form with three studies prior to data abstraction. A third author (KF) verified the accuracy of the data. Each included study was inspected to identify the reported features:

- characteristics of the article (e.g., first author, publication year) and study (e.g., aims, design, ethics, conclusions, limitations)
- the intervention, including the knowledge organizing systems [67] (theories, frameworks, models) underpinning the approach to shared decision making
- the features described in our conceptual framework (see Fig. 1)
- study authors’ explanations of facilitators and/or barriers, outputs, and outcomes.

### Data synthesis

One member of the team (JJ) led the conduct of framework synthesis with three other authors (SO, KF, BH) and the oversight (review processes, discuss, provide guidance) of the team, by 1) becoming familiar with the data, 2) applying the conceptual framework to abstracted data, 3) tabulating and interpreting the findings [68]. Data that did not correspond to the conceptual framework were incorporated as emergent themes. The findings were reflected upon, and discussed among team members to confirm similarities and differences within the data, and to determine associations between themes [69]. Our team engaged in reflection on the objectives of our review to guide interpretations in the conduct of the synthesis.

## Results

### Search results and characteristics

The search identified a total of 5068 studies of which we included nine studies, reported in 10 publications (Supplementary file 5). The studies were conducted in the United States (*n* = 4), Canada (*n* = 3), New Zealand (*n* = 1) and Australia (*n* = 1). The studies were focused on the development and/or evaluation of shared decision making interventions in the areas of Depression (*n* = 1), Cancer screening (*n* = 1), Child welfare (*n* = 2), Any health decision (*n* = 1), Cancer care (*n* = 1), Intimate partner violence (*n* = 1), Tobacco use (*n* = 1), and Arthritis (*n* = 1). Most of the studies were a qualitative design (*n* = 8) and reported some form of Indigenous and academic research partnership (*n* = 7).

The characteristics of included studies are listed in Table 1.

**Table 1:** Characteristics of included studies

Study	Country	Team	Topic	Participant Groups	Number of Indigenous Participants	Age Range (years)	Methodological Framework	Data Collection	Analysis
*Dirks et al., 2018 [70]	US	Indigenous and academic partners	Develop and evaluate a decision support tool with AI/AN, for discussion about depression	AN/AI who will discuss depression management	375	40.7^x^	Qualitative	Survey, focus groups	Thematic analysis
Frerichs et al., 2020 [71]	US	Indigenous and academic partners	Evaluate the impact of the colorectal cancer (CRC) screening decision aid adapted for AI adults on their CRC-related outcomes	AI considering colorectal cancer screening	104	50–75	Pre-post study design, pilot test	Tools with scales	Statistical analysis
Grace et al., 2018 [72]	Australia	Indigenous and academic partners	Examine whether Aboriginal children in child protection services relate and respond to the Kids Say cards	Children in child protection	20	8–18	Qualitative	Interviews	Content analysis of qualitative data
Jull et al., 2015 [73]	Canada	Indigenous and academic partners	Describe the adaptation and usability testing of the Ottawa Personal Decision Guide (OPDG) to support decision making with Indigenous women	Members of an FNIM women’s community who have left situation of violence or impacts of residential schools	19	20–60	Qualitative	Focus groups, interviews	Thematic analysis
Jull et al. 2019 [46]	Canada	Indigenous and academic partners	To tailor and field-test a shared decision-making strategy for use by Inuit in cancer care	Inuit facing decisions about cancer care, peer support workers	13	n/r	Qualitative	Focus groups, interviews	Thematic analysis
Koziol-McLain et al., 2018 [74]	New Zealand	Academic	To test the efficacy of a Web-based safety decision aid (iSafe) for women experiencing IPV	Women making decisions about safety	113	16–59*	Randomized control trial	Tools with scales	Statistical analysis
Marcynyszyn et al., 2012 [75]	US	Indigenous and academic partners	Describes an adapted Family Group Decision Making practice model and its evaluation among Lakota community for child welfare	Decisions about child welfare	n/r	n/r	Qualitative	Observation	Description of the process
Montgomery et al., 2012 [76]	US	Academic, unclear on research relationship with Indigenous partners	Describes the development of curriculum that trains AI/AN youth leaders to plan, write, and design comic books to enhance healthy decision making in tobacco use	Youth in afterschool youth program	6	12–15	Qualitative	Surveys, participant observation, interactive meeting	Description of the process
*#Starks et al., 2015 [77]	US	Indigenous and academic partners	Develop and evaluate an application to engage AI/AN in conversation about depression with healthcare providers	AN/AI who will discuss depression management	36	n/r	Qualitative – case study	Interviews, documents, stakeholder consultations	Stakeholder engagement consisting of meetings, mapping phases, iterative discussions, critical reflection to describe the final DM-DST and project cycle
Umaefulam et al., 2022 [78]	Canada	Indigenous and academic partners	To adapt the Early RA patient decision aid for use with Indigenous patients	Indigenous patients facing RA decisions	16	28–69	Qualitative	Interviews	Thematic analysis

*SDM* shared decision making

*DM-DST* Depression Management Decision Support Tool

*AI* American Indian

*AN* Alaskan Native

*FNIM* First Nations, Inuit, Métis

*IPV* Intimate Partner Violence

*RA* Rheumatoid Arthritis

*PtDA* Patient Decision Aid

^*^One study reported in two publications

^x^ Detail on age range not provided (mean age of 40.7 (SD = 16.2)

^#^data not disaggregated for Indigenous participants

The descriptions of interventions to support the shared decision making process ranged from online/electronic, paper-based, and paper-based plus training, to models of practice to facilitate decision making. The providers of the shared decision making interventions included health care providers, trained decision coaches, coordinators or agency personnel, trained youth and project staff, or trained researchers who were testing the intervention with the intent for use by health care providers. Two interventions were designed to be used without provider involvement. The studies reported the use of the shared decision making intervention prior to meeting the health care provider, during the health care provider meeting, or during a prolonged process before and during making a decision. Most studies (*n* = 8) report knowledge organizing systems (theories, frameworks, models) for the shared decision making intervention as Indigenous only (*n* = 2), Indigenous and Western-oriented (*n* = 3), or Western-oriented only (n = 3).

The characteristics of interventions are listed in Table 2.

**Table 2:** Characteristics of interventions

Author (year)	Brief name and why	What (materials, process) for the SDM intervention	Who provided SDM	How, Where, When, How much	Develop, adapt SDM intervention	Knowledge organizing systems (theory, model, framework)
*Dirks et al., 2018 [70]	Pilot of DM-DST-Evaluate effectiveness of DM-DST for AN/AI	An electronic tool (DM-DST) to engage AN/AI in decision making conversations with healthcare providers	iPad application administered by primary care providers or behavioural health consultants	Participants used 20-min decision support tool prior to meeting healthcare provider at primary care appointment	Application developed with community stakeholders for use by AN community members	Nuka System of Care
Frerichs et al., 2020 [71]	Culturally Adapted Decision Aid -Evaluate CRC screening decision aid adapted for AI	9-min video (decision aid) to prepare AI to participate in CRC screening decisions	Video disseminated through web, prompts viewers to speak with healthcare provider	Participants viewed online video about screening options streamed or downloaded at home	Video developed by researchers for use with AI communities in eastern US	Social cognitive theory, theory of planned behaviours
Grace et al., 2018 [72]	Kids Say Cards-Examine response of children to Kids Say tool	Kids Say cards to facilitate conversation and engagement by children in decision making with child welfare workers	Researchers* use with children *designed to be used by child welfare Workers/Professionals	Researchers chose 10 out of 44 Kids Say cards focus group or individual interview (time not reported) to prompt child(ren) in conversation, at community event	Cards developed by Aboriginal community for Aboriginal children	Winangay knowledge—approach guided by National Health and Medical Research council's values of equality, respect, reciprocity, responsibility, survival, and protection
Jull et al., 2015 [73]	Adapted OPDG (Decision Aid) -Adaptation of OPDG to support decision making by Aboriginal women	A patient decision aid for use by Indigenous women and includes decision coaching	Decision Coach	60–90 min interviews in community setting supported by decision coach to apply tool to sample decision	A generic decision aid adapted by Indigenous and non-Indigenous team with community partnerships and t with Indigenous (FNIM) women in Canada	Ottawa Decision Support Framework, Medicine Wheel framework for shared decision making
Jull et al. 2019 [46]	Not Deciding Alone -Promote the use of SDM by Inuit in Cancer Care	A booklet and training to guide conversations between healthcare providers and clients, to prepare for SDM	Peer support worker trained in decision coaching	Trained peer support worker was matched with community member to read and understand booklet and have conversation using SDM strategy in booklet in community setting	An SDM strategy (booklet, training) developed with Inuit community and service providers, for Inuit	Inuit societal values, Ottawa decision support framework
Koziol-McLain et al., 2018 [74]	iSafe-Test efficacy of web-based safety decision-aid (iSafe) for women experiencing IPV	A web-based decision aid developed for general populations applied with women (includes subgroup of Māori) in New Zealand who experience IPV	Online decision-aid/intervention website	Women in intervention group were provided link and password to website throughout 1-year postbaseline follow-up period to intervention and individualized resources and safety plan; control group offered link and password to website with standardized resources and safety plan Intervention done at home	n/r	Empowerment model and decision science
Marcynyszyn et al., 2012 [75]	Family Group Decision Making -Promote a decision-making practice model for Native American communities that could be used in Child and Family Services	A Māori Family Group Decision Making practice model, implemented with child and family services setting to bring in Lakota ways of building and sustaining family connections	Independent Coordinator, Agency personel	Independent coordinator convenes family group meeting with agency personnel, after initial presentations family group meets on own to work through information given and formulate response and plan, preference is given to family groups plan, referring agency support family to implement plan	A Māori practice model adapted to Lakota knowledge and culture	Māori traditional practices, adapted to include Lakota traditional values and practices
Montgomery et al., 2012 [76]	Native Comic Book Program -Train Native youth leaders to create comic books to enhance healthy decision making	A curriculum for AI/AN youth to design comic books for healthy decision making	Project Staff/Trained youth	2 h sessions over 8-week period during summer after-school program where youth participated in curriculum focused on decision-making and comic book development	A curriculum developed for general youth populations adapted for AI/AN youth	Western-oriented knowledge about shared decision making (model)
*Starks et al., 2015 [77]	DM-DST -Development of depression management decision support tool	Development of DM-DST for AI/AN to integrate patient priorities and needs into depression management decisions	iPad application administered by primary care providers/behavioural health consultants	Smaller pilot test (*n* = 20) had participants go through 20-min decision application	Application developed with community stakeholders for use by AN community members	Nuka System of Care
Umaefulam et al., 2022 [78]	Adaptation of Early RA PtDA -Adapt existing RA PtDA for use by Indigenous patients at point of care	Adaptation of existing paper-based decision aid tool	Displayed at	Cohort 1: 15–45 min in-person interview at clinic and asked to provide feedback when provided with decision aid Cohort 2: 15–45-min video conference or phone interviews about adaptation of decision aid (provided earlier)	An RA decision aid adapted for use by Indigenous people in Canada	Ottawa Decision Support Framework

*SDM* shared decision making

*DM-DST* Depression Management Decision Support Tool

*AI* American Indian

*AN* Alaskan Native

*FNIM* First Nations, Inuit, Métis

*IPV* Intimate Partner Violence

*RA* Rheumatoid Arthritis

*PtDA* Patient Decision Aid

^*^One study reported in two publications

### Study quality: reflections on strengths and relevance

We reflected on the strengths and relevance of the included studies to determine the contributions to shared decision making and Indigenous self-determination [64, 65]. To determine contributions to shared decision making, we reflected on the community relevance, rigour of the methodology, and the strength of the evidence reported in the studies. We found studies to range from medium to high for eight of the nine interventions. Our reflection centred on six areas related to Indigenous self-determination; of the nine studies, only one study (reported in two publications) was found to provide information on all six areas of Indigenous self-determination.

The strengths and relevance of the included studies are reported in Table 3.

**Table 3:** The strengths and relevance of the included studies

Study	Appraisal Score: Low = 6–7 Med = 8–9 High = 10–12	Prioritization	Governance	Relationships	Methodologies	Capacity	Dissemination
*Dirks et al., 2018 [70]; Starks et al., 2015 [77]	High	x	x	x	x	x	x
Jull et al., 2015 [73]	High	x	x	x	x	x	-
Jull et al., 2019 [46]	High	x	x	x	x	x	-
Umaefulam et al., 2022 [78]	High	x	x	x	x	x	-
Grace et al., 2018 [72]	Med	x	x	x	x	x	-
Marcynyszyn et al., 2012 [75]	Med	x	x	x	x	-	x
Frerichs et al., 2020 [71]	Med	-	-	x	-	-	-
Koziol-McLain, et al., 2018 [74]	Med	-	-	-	x	-	-
Montgomery et al., 2012 [76]	Low	-	-	x	-	x	-
**Prioritization:** Research reflects need/priority of people who are the focus of the research			**Governance:** Indigenous research leadership			**Relationships:** Research partnerships	
**Methodologies:** Indigenous paradigm, methods, values			**Capacity:** Researcher, Community partners			**Dissemination:** Sharing to benefit people who are the focus of the research	

^*^One study reported in two publications

We categorized the nine studies into two clusters of studies: a larger cluster that we identified as Indigenous-oriented, and a smaller cluster identified as Western-oriented (Table 4). The six studies in the Indigenous-oriented cluster report on research that reflects Indigenous self-determination and the priorities of people who are the focus of the research [46, 70, 72, 73, 75, 77, 78]. In addition, studies in this cluster report on the use of Indigenous knowledge organizing systems (theories, frameworks, models), with Indigenous-led or partnered governance models. The exception was a study that was conceptualized, developed and conducted by Indigenous scholars who did not explicitly report Indigenous knowledge organizing system [78]. The studies in the Indigenous-oriented cluster met most (at least 5/6) of the CONSIDER statement criteria, with dissemination being the least reported. The exception was one study about an Indigenous approach to evaluation of a shared decision making intervention, that was conducted by an Indigenous community and did not report on capacity building of researchers or community members [75]. Of the three studies in the smaller, Western-oriented cluster [71, 74, 76], none of the studies report on research that reflects the priorities of people who are the focus of the research or Indigenous leadership (governance). These studies reported at most two of the CONSIDER statement criteria. In addition, none of the studies in the Western-oriented cluster report on the use of Indigenous knowledge organizing systems, and only one of the studies reports on an Indigenous partnership.

**Table 4:**
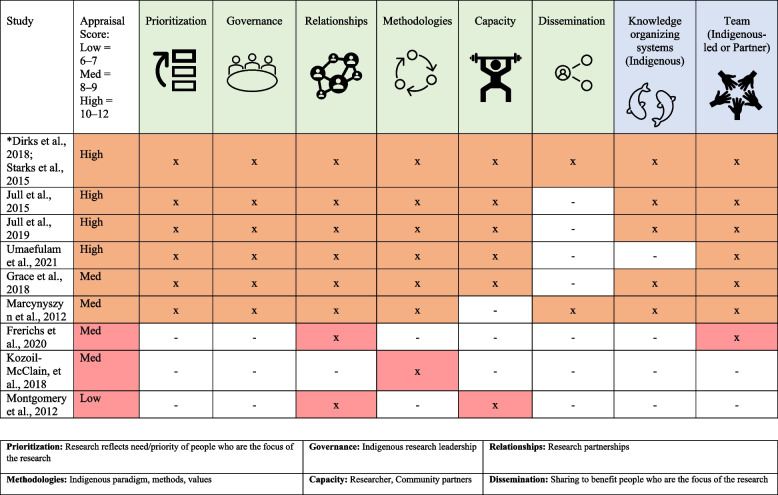
The strengths and relevance of the included studies with knowledge organizing systems and team governance

Indigenous-oriented (orange) = [46, 70, 72, 73, 75, 77, 78]

Western-oriented (red) = [71, 74, 76]

^*^One study reported in two publications

The strengths and relevance of the included studies with knowledge organizing systems and team governance are reported in Table 4.

### Synthesis

**Fig. 3:**
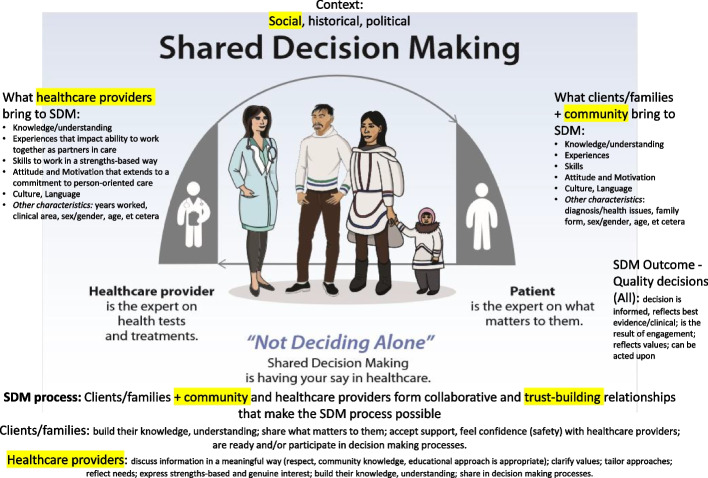
The shared decision making process framework (version 2)

We assessed the included studies in relation to the conceptual framework, and identified four themes, reflected in an updated conceptual framework, The Shared Decision Making Process Framework (version 2) (Fig. 3):

- 1) Where shared decision making takes place impacts decision making opportunities,
- 2) Little is known about the characteristics of health care providers who engage in shared decision making processes,
- 3) Community is a partner in shared decision making,
- 4) The shared decision making process involves trust-building.

#### Theme 1. Where shared decision making takes place impacts decision making opportunities

The first theme relates to the importance of context for shared decision making. All of the included studies report on aspects of the structural determinants of health: that is, the social, historical and political contexts that underpin opportunities for health and wellness in society and reflect the continuing impacts of colonialism in society. These determinants are the basis for understanding the importance of relationships, interconnections, and community [8].

The included studies present the development and/or evaluation of shared decision making interventions as a way to negotiate the impacts of the structural determinants of health or disrupt colonialism in health systems. Place, in particular the built environment, was reported in the studies to reflect the underlying power dynamics in the relationships, and values of people who control the spaces. We define the concept of “space” to mean a physical space and geography (location, form of built environment). The concept of “place” gives meaning to space, as place is space where values are expressed and there are connections to cultural and personal identity [79].

The three studies in the Western-oriented cluster describe the development of interventions to be used in settings preferred by Indigenous participants. Two of these studies specifically identify Western-oriented settings as problematic places and the shared decision making intervention as a way to avoid these settings [71, 74]. For example, one study reports that “Decision aid viewing was not linked to clinical visits, and participants viewed the decision aid privately in their homes or at the location from which they were recruited (i.e., church or senior centre).” [71]. The third study in the Western-oriented cluster reports on the shared decision making intervention as a way to bring decision making to places where traditional and cultural practices are upheld [76].

The six studies in the Indigenous-oriented cluster describe processes to develop and evaluate [46, 72, 73, 75] or to develop and implement [70, 77] shared decision making interventions intended for use within Western-oriented health care systems. For example, a study to determine the acceptability to Aboriginal children of a culturally appropriate tool that facilitates the voices of Aboriginal children and young people in care by supporting professionals to ask good questions and listen. The study identifies the importance of “providing spaces for children and young people to engage requires consideration of the conditions in which they might be comfortable to participate” [72]. Another study describes the development of a stakeholder driven decision support tool to address the issues that included lack of patient preparation to participate with health care providers, and recommendations from health care providers that “…were not in line with patient values and preferences.” [70]. Western-oriented health care settings are built to support Western-oriented values and approaches to health, and are places that are perceived by Indigenous (and other groups) to limit opportunities for person-centred care [80]. The studies in our review present shared decision making interventions as strategies to position Indigenous Peoples in health care settings as decision makers, rather than passive recipients of care.

#### Theme 2. Little is known about the characteristics of health care providers who engage in shared decision making processes

We found that the included studies report little or no data about health care providers and their shared decision making knowledge, experience, skills, attitude and motivation, as well as other characteristics such as culture and language. Relational competencies in shared decision making are defined as those necessary to create an environment for communication and interaction in clinical settings and includes listening to and involving people to the degree that they wish to be involved [81]. Of the nine included studies, five of the studies in the Indigenous-oriented cluster report on the health care providers as the partner involved in the shared decision making relationship as Indigenous [46, 75], non-Indigenous [73], or who were not reported as Indigenous or non-Indigenous [72, 77]. Of these studies, only four report specific information about health care providers in relation to the shared decision making intervention [46, 73, 75, 77].

Of the four studies from the Indigenous-oriented cluster that report specific information about health care providers, two report on the knowledge health care providers have about shared decision making and the health system [46, 73]. All four studies report that the previous experience of health care providers with shared decision making is important to support patients in decision making [46, 73, 75, 77]. Three of the four studies report on health care provider skill (how to support engagement of patients) and attitude (value of patient participation in decisions, a commitment to person-oriented care) [46, 73, 75]. One additional study reports on the experience, skill, and attitude health care providers should bring to be partners in care, as a willingness to listen and to take patient views and experiences seriously [72]. Three of the four studies report on the characteristics of health care providers, that includes culture and language [46, 73, 75]. Two studies report on additional characteristics (e.g. years of work, clinical area, gender, age) of Indigenous (*n* = 5) [46] and non-Indigenous (*n* = 1) [73] health care providers involved with the shared decision making interventions.

The characteristics of participants (patients, health care providers) related to shared decision making, culture/language and other characteristics, are reported in Table 5.

**Table 5:** Characteristics of participants (patients, health care providers) related to shared decision making, culture/language and other characteristics

Author, year	Participants: Patients Health care providers	SDM Knowledge	SDM Experience	SDM Skills	SDM Attitude/Motivation	Culture/Language	Other characteristics (2 or more): e.g., age, sex/gender, occupation
*Dirks et al., 2018 [70]	Clients	x	x	-	-	-	x
	HCPs	-	-	-	-	-	-
Frerichs et al., 2020 [71]	Clients	-	-	-	-	x	x
	HCPs	-	-	-	-	-	-
Grace et al., 2018 [72]	Clients	x	x	-	-	-	x
	HCPs	-	-	-	-	-	-
Jull et al., 2015 [73]	Clients	x	x	x	x	x	x
	HCPs	x	x	x	x	x	x
Jull et al., 2019 [46]	Clients	x	x	x	x	x	x
	HCPs	x	x	x	x	x	x
Koziol-McLain et al., 2018 [74]	Clients	-	-	-	-	x	x
	HCPs	-	-	-	-	-	-
Marcynyszyn et al., 2012 [75]	Clients	x	x	x	x	x	x
	HCPs	-	x	x	-	x	-
Montgomery et al., 2012 [76]	Clients	-	-	-	x	-	-
	HCPs	-	-	-	-	-	-
*Starks et al., 2015 [77]	Clients	x	x				
	HCPs	-	x	-	-	-	-
Umaefulam et al., 2022 [78]	Clients	-	x	-	-	x	x
	HCPs	-	-	-	-	-	-

*SDM* shared decision making, *HCPs* health care providers

^*^One study reported in two publications

#### Theme 3: Community is a partner in shared decision making

The next theme relates that community – meaning the groups with which individuals are connected and hold shared beliefs – is an important partner in health decision making. We found the included studies to identify a role for community as a source of knowledge and support in shared decision making.

All the studies report that the values and knowledge held within community networks support participation of patients and families to make health decisions [46, 70–73, 75–78]. For example, one study describes shared decision making as an engagement process that positions families and communities to “reclaim customary practices and to resolve issues within their wider circle” [75]. In another study the authors reflect on the need to align a curriculum for healthy decision making for youth, with the values of family and community, in addition to the place and personal gifts (skills, knowledge) of people involved [76]. A subset of the included studies describes how to engage community members in partnerships.

Five of the studies from the Indigenous-oriented cluster [46, 72, 73, 75, 78] and one from the Western-oriented cluster [76] report that those who deliver the shared decision making intervention need to understand the community values in which shared decision making will take place. These studies describe the delivery of the shared decision making intervention by members of the community [46, 76], or those who are non-Indigenous and have the knowledge and skills to partner with Indigenous patients and communities [72, 73, 78]. For example, in a study conducted with urban First Nations, Inuit and Metis women, the community members identify the importance of respect for their community traditions and communication styles in shared decision making and “…encouraged the decision coach in a process of learning *with* them as well as *about* them and their decision making needs” [73].

#### Theme 4: The shared decision making process involves trust-building

Studies from the Indigenous-oriented cluster report on factors that enhance research practices with Indigenous Peoples and increase research accountability [65] and trust-building in the development and evaluation of shared decision making interventions (see Table 4). For example, one study describes an approach to shared decision making to overcome intergenerational grief and trauma experienced by the Indigenous communities and that positions families and communities to reclaim customary practices. The study authors report on their approach to engagement of families in decisions that relate to child welfare, to overcome the lack of trust between families, health care providers, and others in the health system [75]. In another example, a team evaluated an intervention called “The Kids Say” that involves the use of cards to prompt conversation with health care providers “…to open the way for young people to share their experiences and be part of decisions that are about their lives” [72]. The study authors report that a relationship of trust is an essential feature of the shared decision making process. Furthermore, the authors indicate that children and young people should not be asked to share their views with health care providers “…unless the relevant adults are prepared to take them [children and youth] seriously and to act on their behalf” [72]. Other studies describe meeting the socio-cultural needs of the community partner, and the role of the shared decision making intervention to help people deal with the colonial history and ongoing negative experiences through engagement and relationship building with their health care providers [46, 78]. The Indigenous-oriented studies reported on processes to support trust-building as an essential feature of shared decision making processes.

We found limited reporting in studies from the Western-oriented cluster on factors to support research accountability and trust-building to enhance research practices with Indigenous Peoples. Instead, these studies focused on interventions that people could use to avoid the negative influences of health systems. For example, a web-based safety decision aid for women facing intimate partner violence was designed to offer women the opportunity to prioritize and plan for safety for themselves and their families. In their discussion, the authors propose that for Maori women who face racism and other significant barriers to service use, the option to use a decision aid outside of the health system is a way to avoid potential racism or discrimination [74]. Another team proposed a patient decision aid that can be used by people who identify as American Indian, to address issues with participation in cancer screening that are the result of mistrust of health care providers and systems [71]. In these examples, the shared decision making interventions are proposed as a way for patients to navigate health systems that are not trusted sources of support.

## Discussion

**Fig. 4:**
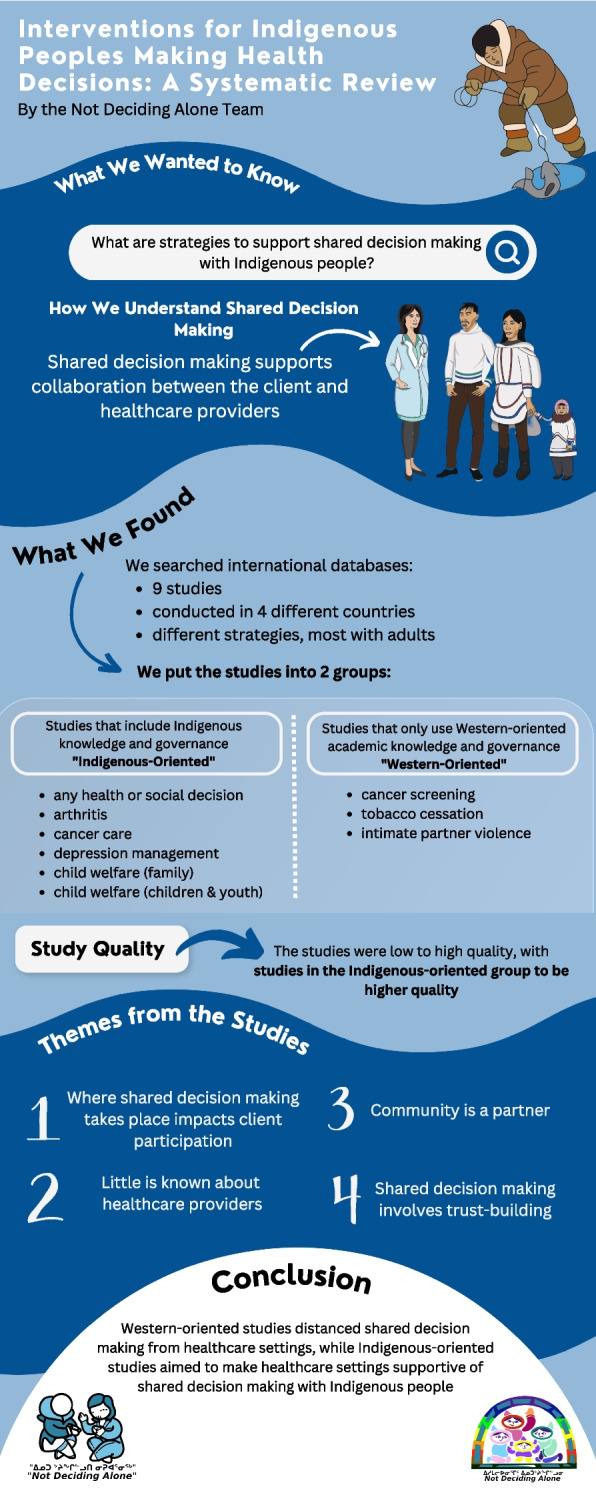
Summary of our study

Our review was conducted to identify interventions about shared decision making for Indigenous Peoples making health decisions, to synthesize evidence and to identify factors (barriers and facilitators) that impact the use of shared decision making interventions (Fig. 4). Here, we identify and describe interventions and contribute to other, previously conducted reviews about interventions that support shared decision making with Indigenous Peoples [31] and groups who experience disadvantage [30]. Our review also contributes to the broader literature about the potential for shared decision making interventions to advance health equity for people who experience limited opportunities to participate in their health decision making [82–84]. Our findings report on examples of shared decision making interventions, and include a conceptual framework to provide a foundation for reflection on shared decision making processes.

We used a conceptual framework to extend consideration of the relational features of shared decision making processes to identify: impacts of place (where shared decision making happens) on opportunities for participation in health decision making; a gap in the reporting about characteristics of health care providers in relation to shared decision making processes; the inclusion of community as a source of knowledge and support and a partner in shared decision making; trust-building as a complex, important and active process integral to shared decision making. We report the findings in an updated conceptual framework that depicts shared decision making processes as highly relational (Fig. 3).

### Extend understanding of shared decision making as a relational process

Western-oriented health care settings are built to support Western-oriented values and approaches to health, and are places that may be perceived by Indigenous Peoples (and other groups) to limit opportunities for person-centred care [80]. The Indigenous-oriented studies in our review present shared decision making interventions as strategies to position patients in health care settings as decision makers in their care.

The Indigenous-oriented studies focused on resources and training to improve discussions between Indigenous participants and health care providers within health systems. These approaches include: training community support workers in shared decision making [46]; training community and agency workers in a family group decision making practice [75]; resources and workshops for supporting professionals’ skills in active and respectful listening [72]; a decision aid and coaching for sharing decisions between patient and health care provider(s) [73]; and a decision support tool to facilitate discussions between patients and their health care providers [70, 77].

In contrast, the Western-oriented studies adopted health promotion approaches for Indigenous participants to support decision making in homes or community settings outside of the health system: to encourage their uptake of cancer screening [71]; to protect themselves from violence and its impact on mental health [74]; and to engage in activities to learn about and 'enhance healthy decision making’ [76]. The findings of our review also highlight the importance of who is present to participate in health decision making with patients and families.

The role of health care providers with shared decision making is highlighted in the literature. In studies with general populations, health care providers have been found to often *not* involve patients in decision making about their care [85, 86], an issue also identified by Indigenous Peoples [16, 60, 87]. Other studies have identified the impacts of health care provider characteristics on shared decision making experiences of patients and families who experience disadvantage in society [88]. Our study highlights a gap in reporting on the characteristics of health care providers involved in the delivery of interventions to support shared decision making. In addition, many of the studies included in our review describe how non-Indigenous health care providers need to foster active relationships with Indigenous community networks to better engage in shared decision making processes with people in the community [46, 72, 73, 75, 76, 78].

Our review describes shared decision making processes as highly relational and trust as a foundation for the relationships. Studies from the Indigenous-oriented cluster report on factors that enhance research practices with Indigenous Peoples and increase research accountability [65] and trust-building in the development and evaluation of shared decision making interventions. Trust in the health care system and interpersonal relationships (between patient and health care providers) are an essential feature of shared decision making processes [89, 90]. We found that all the studies in our review emphasize the importance of active processes of trust-building among patients, families, communities, and health care providers. The focus on trust-building in shared decision making is identified elsewhere in the literature [89, 91, 92], and a feature of research agendas aimed at supporting person-centred care [90, 93].

### High quality research upholds Indigenous self-determination

The findings of our review include examples of research studies that demonstrate how to support Indigenous self-determination in research about shared decision making. The included studies provide examples of high-quality solutions-focused research. Research that is undertaken in collaboration with those who will use or be impacted by the research fosters democratic processes of knowledge coproduction [48], and is an important source of information to develop policies to support patient values and preferences in healthcare decisions [94]. Research partnerships with those who will use or be impacted by the research [95] has been identified as important for successful implementation of shared decision making [96, 97]. Our review identified multiple models for collaborative research with a ‘nothing about us, without us’ approach, a central feature of research that upholds Indigenous self-determination [35]. The conduct of research with the collaboration and governance of Indigenous team members, founded on Indigenous knowledges, means research is more likely to produce evidence that is useful and able to be used by those ultimately impacted by the research [98]. For Indigenous Peoples, colonization, racism, social exclusion and self-determination are important concepts that must be considered in research practices [9]. In our review, Western-oriented studies focused on interventions that people could use to avoid the negative influences of health systems. In contrast, we found the Indigenous-oriented studies sought to change health systems by supporting Indigenous Peoples to participate in their health decision making.

### Limitations and strengths

The limitations and strengths of the review centre on the appropriateness and relevance of reviews to address Indigenous issues [99]. The potential limitations of this review include poor indexing of studies in databases, a lack of tested protocols for conducting systematic reviews in Indigenous health, and the potential for inclusion of more grey literature. In response, given the poor indexing of studies in electronic databases, it is possible that some studies were missed; however, there is transparency in the extensive search strategy. The review is limited by the reality that currently most health research is written, reviewed, and published by non-Indigenous scholars and journals in academic systems that are founded in colonial systems. The important contributions of Indigenous scholars and knowledge systems to health research contexts will become more visible as the capacity for journals to support publication of health research that is equity-oriented improves and with the increasing numbers of Indigenous researchers who extend thinking about how to conduct health research. Our review is not intended as an effectiveness review and involves framework synthesis that is unlikely to substantially change with the inclusion of new evidence [100]; additionally, no new studies were located with the grey literature search. We anticipate using the findings from this review to build grey literature search strategies in another, future review.

The strengths of our review include: 1) the oversight and governance of Indigenous team members to embed Indigenous knowledges and practices in our review processes; 2) the use of a conceptual framework to organize concepts important to shared decision making with Indigenous Peoples; 3) a comprehensive search strategy that was developed with an academic librarian, the use of two independent reviewers at each screening stage, and; 4) Indigenous critical appraisal tools to identify the strengths and relevance of included studies’ contributions to shared decision making and Indigenous self-determination. Additionally, the use of the ENTREQ and PRISMA-Equity guidelines in this review provide a standardized approach to reporting and contributes to building evidence on best standards for systematic reviews supporting health equity [40, 41].

## Conclusions

Health care systems and settings have been identified as places where racism and negative histories exist, with policies that structure routines and behaviour that are not inclusive [80, 101]. While there are examples of health care settings that have been designed to provide culturally appropriate care that include care for Indigenous Peoples [102–104], there are limited descriptions of interventions to support Indigenous Peoples to participate in health decision making. Shared decision making tools and approaches are complex interventions that are developed to facilitate shared decision making process with people who span health systems, from patients and families to policy makers [105].

Our review provides examples of how to develop and evaluate shared decision making interventions with Indigenous Peoples. It extends thinking beyond Western-oriented conceptions of shared decision making interventions. Overall, Indigenous-oriented studies sought to make health care systems more amenable to shared decision making for Indigenous Peoples, while Western-oriented studies distanced shared decision making from health care settings. Our findings demonstrate that it is possible to do high quality research to develop and evaluate shared decision making interventions with potential to support Indigenous Peoples to participate in their health decisions. Further studies that are solutions-focused and support Indigenous self-determination are needed.

### Supplementary Information

**Additional file 1.**

**Additional file 2.**

**Additional file 3.**

**Additional file 4.**

**Additional file 5.**

## Data Availability

All data generated or analysed during this study are included in this published article and its supplementary information files.

## Footnotes

1 Indigenous communities, Peoples and nations have a historical continuity with pre-invasion and pre-colonial societies that developed on their territories and consider themselves distinct from other sectors of society that now prevail on those territories (1,2) In Canada, there are 3 distinct Peoples with unique histories, languages, cultural practices and spiritual beliefs: First Nations, Inuit and Métis (3). We capitalize Indigenous Peoples to recognize Indigenous Peoples as Nations.
