# Supplementary material for: Interventions for Indigenous Peoples making health decisions: a systematic review
Source: Arch Public Health. 2023 Sep 27;81:174. doi: 10.1186/s13690-023-01177-1 (PMC10523645; doi:10.1186/s13690-023-01177-1)
Supplement: Supplementary file 3 — Additional file 3. [file 13690_2023_1177_MOESM3_ESM.docx]

Supplementary file to accompany Figure 1 The Shared Decision Making Process Framework:

We devised an organizing framework for examining shared decision-making with Indigenous Peoples making health decisions, with key distinguishing features (107): as:

- Context: social (attitudes or approaches) historical (events or circumstances) and political (policy or organizational rules that institutionalize interactions)
- the patient/family: (diagnosis/health issues, family form, sex/gender, age, et cetera), and what they and their families bring to shared decision making (knowledge/understanding, experiences, skills, attitude and motivation, culture, language,
- the health care provider(s): (profession, years worked, clinical area, sex/gender, age, et cetera) and what they bring to shared decision making: knowledge/understanding, experiences that impact ability to work together as partners in care, skills to work in a strengths-based way, attitude and motivation that extends to a commitment to person-oriented care, culture, language,
- Shared decision making process: relationship between clients/families and health care providers

*Clients/families*: build their knowledge, understand; share what matters to them; accept support, feel confidence (safety) with health care providers; are ready and/or participate in decision making processes.

*Health care provider(s)*: discuss information; clarify values; tailor approaches; reflect needs; express strengths-based and genuine interest; build their knowledge, understanding; share in decision making processes.

- Shared decision making outcomes that lead to quality decisions: decision is informed, reflects best evidence/clinical expertise; is the result of engagement; reflects values; can be acted upon.
