# Supplementary material for: Interventions for Indigenous Peoples making health decisions: a systematic review
Source: Arch Public Health. 2023 Sep 27;81:174. doi: 10.1186/s13690-023-01177-1 (PMC10523645; doi:10.1186/s13690-023-01177-1)
Supplement: Supplementary file 4 — Additional file 4. [file 13690_2023_1177_MOESM4_ESM.docx]

Supplementary file 3. Search Strategy

Indians, North American/ 14695

2 Inuits/ 3964

3 inuit*.mp. 4752

4 Health Services, Indigenous/ 3549

5 aborigin*.mp. 10485

6 indigenous.mp. 37602

7 metis.mp. 406

8 first nation*.mp. 5198

9 amerindian*.mp. 2046

10 off reserve.mp. 105

11 on reserve.mp. 212

12 (Native adj1 (man or men or women or woman or boy* or girl* or adolescent* or youth or youths or person* or adult* or people* or Indian* or Nation or tribe* or tribal or band or bands)).mp. 2143

13 exp American Native Continental Ancestry Group/ 22065

14 maori*.mp. 3729

15 eskimo*.mp. 1560

16 aleut*.mp. 1038

17 american indian*.mp. 7249

18 pacific islander*.mp. 4031

19 or/1-18 79177

20 Choice Behavior/ 33614

21 Decision Making/ 99336

22 decision support*.mp. 41158

23 decision making.mp. 234813

24 decision support techniques/ 21430

25 (decision adj3 aid*).mp.7584

26 (decision adj3 tool*).mp. 7582

27 informed consent.mp. 66404

28 ((personal or interpersonal or individual or parent*) adj1 (decision* or choice* or preference*)).mp. 8522

29 ((patient* or consumer* or client*) adj1 (decision* or choice* or preference*)).mp. 30095

30 informed choice*.mp. 2785

31 informed decision*.mp.8625

32 engagement.mp. 77073

33 consumer health.mp. 6031

34 Health Literacy/6961

35 shared decision*.mp. 10713

36 or/20-35 467802

37 19 and 36 2469

38 limit 37 to yr="2012 -Current" 1797
