# Supplementary material for: Interventions for Indigenous Peoples making health decisions: a systematic review
Source: Arch Public Health. 2023 Sep 27;81:174. doi: 10.1186/s13690-023-01177-1 (PMC10523645; doi:10.1186/s13690-023-01177-1)
Supplement: Supplementary file 5 — Additional file 5. [file 13690_2023_1177_MOESM5_ESM.docx]

Studies from databases/registers **(n = 5068)**

References from other sources **(n = 134)**

**Identification**

Included studies ongoing **(n = 0)**

Studies awaiting classification **(n = 0)**

Studies included in review **(n = 10)**

Studies excluded **(n = 3994**

Studies not retrieved **(n = 0)**

Studies assessed for eligibility **(n = 201)**

Studies sought for retrieval **(n = 201)**

Studies screened **(n = 4195)**

Studies excluded **(n = 191)**

Not about shared decision making (n = 116)

Not a Study (Commentary, Editorials, Protocols)

(n = 18)

Wrong population or does not identify Indigenous people in study as distinct group (n = 4)

Intervention does not involve a decision or does not involve a decision that affects health (n = 53)

Duplicate references removed **(n = 1007)**

**Screening**

**Included**
